# Human and economic impacts of natural disasters: can we trust the global data?

**DOI:** 10.1038/s41597-022-01667-x

**Published:** 2022-09-16

**Authors:** Rebecca Louise Jones, Debarati Guha-Sapir, Sandy Tubeuf

**Affiliations:** 1 Institut de Recherche Santé Société (IRSS), Faculté de Santé Publique, Université catholique de Louvain, 1200 Woluwe-Saint-Lambert, Brussels, Belgium; 2 Institut de Recherches Économiques et Sociales (IRES), Université catholique de Louvain, 1348 Louvain-la-Neuve, Belgium; 3 John Hopkins Bloomberg School of Public Health, Baltimore, Maryland 21205 USA

**Keywords:** Natural hazards, Databases

## Abstract

Reliable and complete data held in disaster databases are imperative to inform effective disaster preparedness and mitigation policies. Nonetheless, disaster databases are highly prone to missingness. In this article, we conduct a missing data diagnosis of the widely-cited, global disaster database, the Emergency Events Database (EM-DAT) to identify the extent and potential determinants of missing data within EM-DAT. In addition, through a review of prominent empirical literature, we contextualise how missing data within EM-DAT has been handled previously. A large proportion of missing data was identified for disasters attributed to natural hazards occurring between 1990 and 2020, particularly on the economic losses. The year the disaster occurred, income-classification of the affected country and disaster type were all significant predictors of missingness for key human and economic loss variables. Accordingly, data are unlikely to be missing completely at random. Advanced statistical methods to handle missing data are thus warranted when analysing disaster data to minimise the risk of biasing statistical inferences and to ensure global disaster data can be trusted.

## Introduction

As the global effects of climate change are felt more intensively, so too are the human and economic consequences of catastrophic disaster events. In 2020 alone, disaster events attributed to natural hazards affected approximately 100 million people, accounted for an estimated 190 billion US$ of global economic losses and resulted in 15,082 deaths^1,2^. In light of COP-26 and recent topical events including, but certainly not limited to, the Haitian Earthquake (2021), Central European Floods (2020) and Australian Bushfires (2020), a renewed urgency has been granted to the research of, preparedness to and mitigation of disasters attributed to natural hazards.

Comprehensive historical data held in disaster databases are central for numerous purposes across both the public and private domain to inform emergency disaster relief management; configure catastrophe risk assessment models; and conduct cost-benefit analyses of disaster risk reduction policies^3^. However, inconsistencies in the reporting of disaster events and methodological difficulties quantifying their impacts, mean that disaster databases are prone to gaps in data availability^3,4^. As a result, the scope and reliability of statistical inferences which can be made from disaster data are reduced^5^. Systematic reporting of disaster events is required to minimise missing data. However, to achieve this is a major challenge. Instead, the use of valid and often complex statistical methods to account for missing data are required^6,7^.

Missing data is a common issue across all research areas. Standard practices are adopted in randomised clinical trials to combat patient attrition^8,9^ and in survey-based observational studies to handle unit- and item-non response^10,11^. However, within the disaster literature, there is little insight as to how missing data should be handled.

To date, there are six disaster databases which have global coverage: the Emergency Events Database (EM-DAT); NatCatSERVICE; Sigma; GLIDE; GFDRR; and BD CATNAT Global^12^. This analysis utilises EM-DAT data alone, as it is the only publicly available, global disaster database and is widely cited; an initial search of the terms: ‘EM-DAT’, ‘CRED’, ‘Emergency Events Database’ and ‘International Disaster Database’ in Google Scholar returned 21,000 search results spanning numerous disciplines. EM-DAT was founded in 1988 by the *Université catholique de Louvain* (Belgium) with support from the United States Agency for International Development (USAID), the World Health Organisation (WHO) and the Belgium Government^13^. Data are collated from sources including the United Nations, reinsurance firms, research institutions and the press. As well as reporting the occurrence of major disastrous events attributed to natural, technological and complex hazards, its meta-data captures the human and economic impacts to assist national and international humanitarian action.

In this study, we conduct a missing data diagnosis to assess the extent of missing data in EM-DAT and to identify observable factors associated with missingness. We restrict our analysis to disaster events attributed to natural hazards occurring between the years 1990 and 2020. In addition, through a review of highly-cited empirical literature utilising EM-DAT data, we illustrate how missing data has previously been dealt with. We conclude by providing a discussion on the potential methods to handle missing data within disaster databases.

## Results

### The state of missing data in EM-DAT

**Fig. 1:**
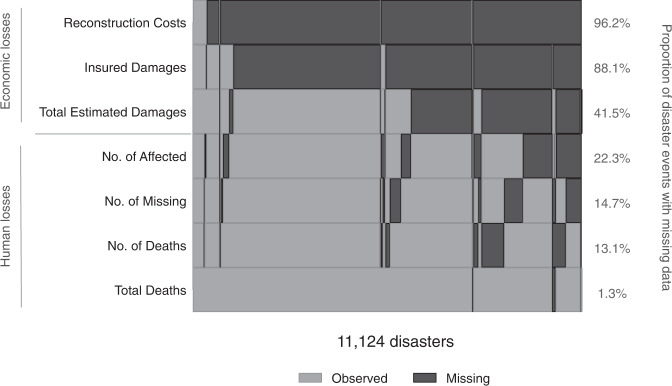
Missing data patterns and proportions of missing data for key human and economic loss variables in EM-DAT across all disaster events attributed to natural hazards and occurring between 1990 and 2020. Black shading denotes missing data for one or more disaster events; grey shading denotes observed data. Disaster events are ordered along the x-axis by the STATA default: from the least to the most missing across the variables analysed. The proportion of missing data for each variable, given as a percentage of the total data, is shown to the right-hand side of the figure. Variables are presented in descending order of missingness. Human loss variables include No. of Affected, defined as the number of people requiring immediate assistance during a period of emergency; No. of Missing, defined as the number of people whose whereabouts is unknown and who are presumed dead; No. of Deaths, defined as the number of people who lost their lives as a result of the disaster event; and Total Deaths, defined as the sum of No. of Affected and No. of Deaths. Economic loss variables include Reconstruction Costs, defined as the costs incurred due to the replacement of lost assets and the implementation of disaster mitigation measures; Insured Damages, defined as economic losses born by the insurance sector; and Total Estimated Damages, defined as a value of all economic losses directly or indirectly related to the disaster event.

The advent of digital technologies in academia and research from the 1980s initiated a proliferation in the collection of data and subsequently, the adoption of data governance and data quality tools to ensure its reliability^14^. In the disaster space, technological advances in disaster surveillance and a progressive global agenda towards standardising disaster reporting, embedded in the 2015–2030 Sendai framework^15^, favour a climate for complete data reporting. Despite this, there was a high proportion of missing data in EM-DAT for disaster events attributed to natural hazards occurring between 1990 and 2020 (Fig. 1). This was particularly evident for the reporting of economic losses: data were missing for 96.2% of disaster events on reconstruction costs, for 88.1% on insured damages and 41.5% on total estimated damages. In the three months following a disaster event, when EM-DAT collates the majority of its data, precise information on reconstruction costs and insured damages is likely to be sparse. Reporting of human losses were more complete, with proportions of missing data ranging from 1.3% to 22.3%. Given that the volume of external disaster aid hinges predominantly on death tolls and injury counts, this finding is unsurprising. Of note, there were substantial inconsistencies in the reporting of both human and economic losses, where in both cases, aggregate variables (total deaths and total estimated damages) were better informed. In particular, missing data on total deaths were negligible (1.3%). Hence, in this case, there is little risk of missing data biasing statistical inferences.

It is important to distinguish not only the extent, but the pattern of missing data to inform the complexity of missingness^16^. For a small proportion of disaster events, data were missing across all variables analysed, denoted in Fig. 1 by the black, continuous vertical band along the right-hand side. More commonly, data were observed for at least one human loss variable and one economic loss variable. For human losses, missing information may be informed through the interdependence of human loss variables; EM-DAT defines total deaths as the sum of the number of people affected and the number of deaths^17^. Whereas for economic losses, there is no such interdependence across economic loss variables. EM-DAT defines total estimated damages as a value of all the economic losses directly or indirectly related to the disaster event, which does not necessarily include reconstruction costs. Missing data occurred sporadically throughout the dataset for each human loss variable, demonstrating an intermittent missing data pattern. However, across the economic loss variables, missing data patterns were less sporadic. For instance, data on reconstruction costs and insured damages were largely missing throughout, even when data on total estimated damages were informed.

### Explaining missing data

Logistic regression analysis was used to identify whether the probability of data to be missing was dependent on observed data within EM-DAT^5,18^. Accordingly, it informs the nature of the missing data mechanism, specifically whether the mechanism of missing data deviates from Missing Completely At Random (MCAR). Data which are MCAR, that is, their probability to be missing is independent of observed and unobserved data, can be excluded from analyses with little risk of introducing bias. Conversely, if the missing data mechanism deviates from MCAR, the deletion of missing values may bias statistical inferences. In this case, more advanced methods are necessary to account for data gaps^6^. Here, we restricted our logistic regression analysis to model the probability of missingness within the variables: total estimated damages, the number of people affected, the number of people missing and the number of deaths (Supplementary Table 1). Methods to account for missing data are viable only for partially incomplete data^5^. In the case of reconstruction costs and insured damages, where data were almost entirely incomplete in our dataset (96.2% and 88.1% missing respectively) little can be done during data analysis to account for missingness. Hence, these variables were not investigated in the logistic regression analysis. The opposite was true for total deaths; the proportion of missing data on total deaths was negligible (1.3%). This precludes logistic regression analysis due to insufficient variation in the outcome variable, the probability to be missing. As a result, the variable total deaths was not investigated in the logistic regression analysis.

The observable data partially explained the probability of total estimated damages to be missing, revealed by a sizeable pseudo-R^2^ value (pseudo-R^2^ = 0.416) (Supplementary Table 1). Missing data on the number of people affected and the number of deaths were explained less by the observed data (pseudo-R^2^ = 0.206 and pseudo-R^2^ = 0.188 respectively) (Supplementary Table 2). This agrees with the intermittent missing data pattern identified for these variables (Fig. 1). The observed data explained much of the probability to be missing for the variable, the number of people missing (pseudo-R^2^ = 0.621) (Supplementary Table 2). However, a large number of disaster events (n = 6,218) were omitted from the analysis by STATA, by default, due to occurring in years which perfectly predicted the probability to be missing. As a consequence, the precision of the parameter estimates is reduced, which requires the results to be interpreted with caution.

A number of observable factors in EM-DAT were statistically significantly (p < 0.05) associated to the probability of total estimated damages to be missing: the year the disaster occured, the income-group classification of the affected country, disaster severity and disaster type (Supplementary Table 1). Disaster events occurring after the year 2002 and in lower-income countries were positive predictors of the probability of data on total estimated damages to be missing. In addition, disaster severity, estimated by the natural logarithm of total deaths, and disaster types having prolonged effects including droughts, epidemics and extreme temperature events were also positive predictors of missingness.

The observed predictors of missingness differed for human loss variables. In contrast to missingness within the variable total estimated damages, the probability of data to be missing for the variables: the number of people affected and the number of deaths were statistically significantly (p < 0.01) lower for disasters which occurred in lower-income countries relative to high-income countries. Lower-income countries are the predominant recipients of international disaster aid. This result suggests that disaster aid incentivises more complete reporting of human losses. The influence of disaster type on the probability to be missing was largely heterogeneous across each variable analysed. In contrast, associations between the year the disaster occurred and the probability of data to be missing were similar across human and economic loss variables, with a higher probability of missingness for disaster events occurring after 2002.

### Shortfalls of the current literature

The absence of published, standard procedures to account for missing data in disaster databases has set a precedent for a general lack of consideration of the issue in the empirical literature. We exemplify this by reviewing the top-20 most cited empirical studies utilising EM-DAT as a primary or secondary data source (Supplementary Table 3).

Eight studies neglected to mention missing data. Of the studies that did, missing data were often considered more broadly under the remit of data availability. With the exception of Brooks, Adger and Kelly^19^ who assessed missing data in-depth in an accompanying paper, mention of missing data or data availability more generally, was limited to one or two sentences.

All disaster events meeting at least one of the following inclusion criteria are included in EM-DAT: i) ten or more people reported dead; ii) 100 or more people affected, injured or homeless; iii) a declaration of a state of emergency by the affected country, or an appeal for international assistance. Accordingly, for disaster events which meet these criteria, data on disaster occurrences are entirely observed in EM-DAT. As such, the extent to which missing data need be considered is dictated by the type of EM-DAT data utilised in empirical studies, namely whether data are utilised on disaster occurrence alone, or on the consequences.

Statistical methods utilised during data analysis typically presume complete information for all variables specified in the model. Therefore, assumptions on the mechanism of missing data are made, whether explicitly or not. Neither the publication year, nor the number of times a paper had been cited affected the approach taken to handle missing data. Of the studies reviewed, half attempted to account for missing data. However, the approaches employed were mostly *ad-hoc* with no statistical grounding. In six studies, authors restricted their analysis to variables or subsets of data with improved data availability. In addition to restricting the scope of their analysis, Brooks, Adger and Kelly^19^ imputed zero values in place of missing values. However, this approach may heavily skew the distribution of data. Dilley *et al*.^20^ aggregated mortality estimates over all disaster events attributed to a natural hazard between 1981 and 2000. This method intends to reduce the relative impact of missing data. Nevertheless, it also compromises the precision of analyses. Barredo^21^ and Doocy *et al*.^22^ supplemented EM-DAT data with data from alternative sources in an attempt to generate a more complete dataset. However, merging of datasets can be time-consuming and can itself introduce ‘file matching’ biases if datasets are heterogeneous in their structure or content^16^. Yang^23^ utilised mean imputation whereby missing values are imputed with a single, unconditional mean of the observed values. Nevertheless, this method is generally not recommended, as the uncertainty in the predicted values due to missing data are not adequately reflected^24^.

### Handling missing data in disaster databases

Identifying a suitable approach to handle missing data will depend on the characteristics of the dataset, the variables of interest and the intended data analysis. Regardless, the choice of missing data method should be grounded on plausible assumptions about the missing data mechanisms as informed through a diagnosis of the missing data. In Supplementary Table 4, we describe conventional and advanced missing data methods which are relevant to handling missing data within disaster databases. An exhaustive review of the various missing data methods is beyond the scope of this paper and can be found elsewhere, see Allison^24^, Graham^25^ and Graham, Patricio and Allison^26^.

Two broad approaches to handle missing data exist: deletion and imputation. Deletion methods, which are so-called ‘quick-fix’ methods, include column deletion, Complete Case Analysis (CCA) and Available Case Analysis (ACA). However, these methods can exclude a large proportion of the original dataset when a high proportion of missing data exists. In addition, deletion methods generally rely on missing data to be MCAR. Hence, when the mechanism of missingness deviates from this assumption, these methods can do more harm than good, biasing statistical inferences reported by studies^7^. Imputation-based methods are of two main varieties: single imputation and multiple imputation. Single imputation methods, such as mean imputation and regression-based imputation replace missing values with a single, predicted value estimated from the observed data. In contrast, multiple imputation generates a range of predicted values through multiply imputed datasets and thus, adequately reflects the uncertainty in the predicted values due to missing data^26^. Other advanced missing data methods include inverse probability weighting, maximum likelihood and Bayesian simulation. In addition, missing data methods can be combined. For instance, Bayesian techniques can be incorporated into the first step of multiple imputation.

## Discussion

Neglecting the issue of missing data exposes studies which inform regional, national and international disaster policies to biases and compromises their effectiveness. The complete, peer-reviewed reporting of disaster events through strengthened field-level data quality remains the eventual goal. In the meantime, given the data available, we argue that to achieve reliable evidence-based disaster policy, it is crucial that missing data is appropriately diagnosed and accounted for in the empirical literature.

This analysis is one of the first to reveal deviations from the assumption of MCAR for missing data on key human and economic loss variables in EM-DAT. Consequently, methods to handle missing data which are MAR should be considered. Deviations from the assumption of MAR, namely that missing data are Missing Not At Random (MNAR) and are thus dependent on unobserved data, cannot be explicitly tested. Instead, sensitivity analyses should be performed to test the robustness of parameter estimates to different assumptions about the mechanisms of missing data. Advanced missing data methods such as multiple imputation, maximum-likelihood and Bayesian simulation can be adapted to account for missing data which are MNAR.

In addition, this analysis highlights key limitations of EM-DAT data. Despite technological advances in disaster surveillance and general progress in data collection, an increase in the proportions of missing data since 2002 suggest shortfalls in current data quality procedures. More-so, the large disparities in the reporting of human losses compared to economic losses points to a wider issue in the types of data collected. Count data in physical units (number of deaths, number of people affected etc.) is principally used to portray human losses, whereas monetary estimates (reconstruction costs, insured damages etc.) are used to denote economic losses. Direct inference of monetary estimates is not straightforward. A two-stage valuation approach may therefore be more suitable and limit the extent of missing data on economic losses. In this case, count data, for instance the number of buildings damaged, the number of days without electricity, would primarily be used to evaluate economic losses in disaster databases. Standard economic valuation methods could then be applied, where necessary, to transform these values into monetary estimates.

By examining the global disaster database EM-DAT, this paper highlights shortfalls in the quality and use of disaster data which are rarely considered. Limitations in the quality of disaster databases arising from data collection procedures have been explored in previous literature^3,4,27,28^. In particular, limitations in the availability of data on low-intensity disaster events due to the inclusion criteria specified by CRED are well known. Instead, this paper contributes a different perspective to the existing literature; given the data collected, to what extent is there an issue of missing data and how should it be handled to ensure global disaster data can be trusted? In this respect, we aim to prompt the reader to apply a critical eye when assessing global disaster data, rather than implicitly trust its reliability.

The extent, complexity and mechanisms of missing data will vary according to the data analysed. Because of this, we are unable to prescribe a single approach to handle missing data within disaster databases. Instead, we advocate for analysts to consider missing data on an individual study basis. Although this article is a step in the right direction, a considerable research effort is still required to inform how missing data within disaster databases should be handled. In particular, the mechanisms of missing data in EM-DAT, and other disaster databases, should be more clearly identified. This would allow data limitations to be better informed and the performance of statistical methods, grounded on plausible assumptions about the mechanisms of missing data, to be tested.

## Methods

### Missing data diagnosis

As per procedures initially employed by Faria *et al*.^18^ and Gabrio *et al*.^29^, but adapted to the context of disaster databases, this missing data diagnosis comprised three steps; i) a description of the proportions of missing data; ii) an analysis of the patterns of missing data; and iii) a logistic regression analysis to assess associations between the probability to be missing and observed values in the dataset. The dataset was restricted to disasters attributed to natural hazards and occurring between the years 1990 and 2020. Data analysis was carried out in STATA (version 16.1) and graphics were produced using the scheme ‘cleanplots’^30^. The code used for the data analysis can be found in the supplementary material (Supplementary Material).

The proportion of missing data was computed across all variables using the command ‘mdesc’^31^. Missing data patterns were produced using the command ‘misspattern, novarsort’^32^. Due to computational limits attributed to a large number of observations for each variable, the option ‘noidsort’ could not be applied. Accordingly, observations were arranged by the default: from the least to the most missing across the variables of interest. Missing data patterns were produced for all human loss variables in the dataset (number of people affected, number of people missing, number of deaths and total deaths) and select economic loss variables (reconstruction costs, insured damages and total estimated damages). While individual direct, indirect, sectoral and infrastructural losses were included in the dataset, these were omitted from visualisations of missing data patterns as they were rarely informed.

Statistical analyses, including the t-test, Pearson’s chi-squared test, Little’s MCRA test and binary probability models, are used to test the assumption that missing data is MAR. That is, missing data can be explained by observed values in the dataset^18^. Logistic regression analysis was used in this study to model the probability of missingness for a given disaster event *i*, conditional on a vector of observable predictors of missingness (*X*_*i*_) (Eq. 1). The outcome variable (*y*_*i*_) was therefore a dichotomous missing data indicator taking value 1 if data were missing, or 0 if data were informed. Missing data indicators were generated for the variables: total estimated damages, number of people affected, number of people missing and number of deaths.

1 $$\left({y}_{i}\,{\rm{| }}\,{X}_{i},{\varepsilon }_{it}\right)=\Pr \left({y}_{i}=1\,{\rm{| }}\,{X}_{i},{\varepsilon }_{it}\right)=F\left({X}_{i}^{{\prime} }\beta \right)$$

with *ε*_*it*_ denoting the error term and *F*(·) denoting the cumulative distribution function (cdf) which takes a standard logistic distribution (Eq. 2) in logistic regression analysis.

2 $$F\left({X}_{i}^{{\prime} }\beta \right)=\frac{1}{1{+exp}^{-{X}_{i}^{{\prime} }\beta }}$$

Observed predictors of missingness (*X*_*i*_) included disaster type, a categorical variable denoting the type of natural disaster, omitting the disaster type flood as the reference to control for multi-collinearity; income-classification of the affected country, omitting high-income as the reference category; and the year the disaster occurred, between 1990 and 2020, included in the model as a categorical variable with the year 1990 omitted as a reference. Reference categories were selected due to being the most frequently observed in the dataset. The year the disaster occurred was included in the model as fixed effects to assess both the trend in missing data over time and the individual threshold effects across each year. Disaster severity, estimated by the logarithm of total deaths, was also included in the model specification when the probability of total estimated damages to be missing was assessed. Otherwise, disaster severity was excluded from the model due to being highly correlated with the variables: number of people affected, number of people missing and number of deaths. Total deaths was log transformed to account for the left-skewed, binomial distribution, arising from the high proportion (60%) of zero reported deaths in the dataset. Observed predictors of missingness were selected based on a step-wise approach.

Logistic regression analysis was estimated by maximum likelihood estimation (Eq. 3) using the command ‘logistic’ for all disaster events having complete information on the predictors of missingness. Marginal effects were computed at the mean using the command ‘mfx’ to enable interpretation of the magnitude of effect in the probability scale.

3 $$LogL={\sum }_{i}\left\{\left(1-{y}_{i}\right)log\left(1-F\left({X}_{i}^{{\prime} }\beta \right)\right)+{y}_{i}log\left(F\left({X}_{i}^{{\prime} }\beta \right)\right)\right\}$$

The robustness of our results was assessed using a probit model and a conditional (fixed-effect) logit model. The latter was used to test for potential ‘incidental parameter’ bias arising from the inclusion of individual fixed effects.

### Review of the literature

Disaster databases are highly prone to missingness. Despite this, little insight exists as to how missing data should be handled in this context. To inform previous approaches to handling missing data in disaster databases, empirical studies utilising EM-DAT as a primary or secondary data source were reviewed. As this review was conducted for illustrative purposes only, we sought only prominent, highly-cited studies since these typically shape the standards for subsequent research. Studies were identified through electronic database searches of Web of Science, Scopus, PubMed and Google Scholar on 01/06/2022 using the key search terms: ‘EM-DAT’, ‘Emergency Events Database’, ‘International Disaster Database’ and ‘CRED’. Only papers considered to be empirical in nature and published between 1990 and 2022 were considered. No language restrictions were applied. Of the initial 2,127 search results, 421 papers were deemed to meet the inclusion criteria. Of these, the top-20 most cited papers were identified. For each paper, data were extracted on the use of EM-DAT data, the consideration given to missing data and the approach taken to handle missing data^33–47^.

## Supplementary information

Supplementary material Table of contents

Results of logistic regression analysis to test associations between the probability of data to be missing on total estimated damages and observable data in the Emergency Events Database (EM-DAT)

Results of logistic regression analysis to test associations between the probability of data to be missing on select human loss variables and observable data in the Emergency Events Database (EM-DAT)

Consideration of missing data in pivotal empirical studies utilising the Emergency Events Database (EM-DAT)

Reference List

Glossary of conventional and advanced missing data methods

STATA code

## Data Availability

The data utilised in this analysis were acquired from the Emergency Events Database (EM-DAT). Data from EM-DAT is publicly and freely available to download from: https://public.emdat.be/. Restrictions apply to the dataset used in this analysis, which included additional data from that freely available. This was acquired by the authors on a direct request to the Centre for Research on the Epidemiology of Disasters (CRED). Data is however available from the corresponding author upon reasonable request and with the permission of CRED, which can be obtained via written request to Ms. Regina Below, the EM-DAT database manager, at regina.below@uclouvain.be.

## Code availability

The code used to generate the results of this analysis are available as supplementary material. Data analysis was carried out in STATA (version 16.1).
