# Supplementary material for: Human and economic impacts of natural disasters: can we trust the global data?
Source: Sci Data. 2022 Sep 16;9:572. doi: 10.1038/s41597-022-01667-x (PMC9481555; doi:10.1038/s41597-022-01667-x)
Supplement: Supplementary file 1 — Supplementary material Table of contents [file 41597_2022_1667_MOESM1_ESM.docx]

# **Supplementary material**

**Table of contents**

Supplementary Table 1

Supplementary Table 2

Supplementary Table 3

Reference List

Supplementary Table 4

STATA code
