# Supplementary material for: Human and economic impacts of natural disasters: can we trust the global data?
Source: Sci Data. 2022 Sep 16;9:572. doi: 10.1038/s41597-022-01667-x (PMC9481555; doi:10.1038/s41597-022-01667-x)
Supplement: Supplementary file 2 — Results of logistic regression analysis to test associations between the probability of data to be missing on total estimated damages and observable data in the Emergency Events Database (EM-DAT) [file 41597_2022_1667_MOESM2_ESM.pdf]

Observable Characteristic	Variable	% of missing data	Coefficient +	SE
		Total Estimated Damages		
	Observations (n)	8,286		
	Pseudo R^2	0.416		
Country Income Group	Low-income	62.7	0.432 ***	0.026
	Lower-middle-income	45.4	0.243 ***	0.020
	Upper-middle-income	36.7	0.095 ***	0.019
	High-income (ref.)	31.2	-	-
Disaster Severity	Log of total deaths	-	- 0.080 ***	0.004
Natural Disaster Type	Drought	62.0	0.700 ***	0.018
	Earthquake	32.4	- 0.083 ***	0.021
	Epidemic	51.0	0.472 ***	0.028
	Extreme Temperature Event	61.7	0.536 ***	0.030
	Flood (ref.)	46.0	-	-
	Landslide	50.6	0.291***	0.036
	Storm	28.0	- 0.147 ***	0.013
	Volcanic Activity	41.7	0.089	0.112
	Wildfire	21.6	- 0.139 ***	0.027
	Other disasters	45.6	0.302 **	0.149
Year	1990 (ref.)	13.9	-	-
	1991	16.2	- 0.158 **	0.053
	1992	14.7	- 0.175 **	0.053
	1993	12.4	- 0.137 **	0.065
	1994	5.9	- 0.133 **	0.063
	1995	4.7	- 0.208 ***	0.039
	1996	22.7	0.197 **	0.092
	1997	12.7	- 0.099	0.061
	1998	10.2	- 0.176 ***	0.042
	1999	8.2	- 0.197 ***	0.035
	2000	4.6	- 0.267 ***	0.017
	2001	7.4	- 0.233 ***	0.026
	2002	10.2	- 0.196 ***	0.035
	2003	39.8	0.389 ***	0.078
	2004	57.3	0.596 ***	0.045
	2005	71.4	0.664 ***	0.031
	2006	77.1	0.682 ***	0.026
	2007	70.4	0.673 ***	0.029
	2008	72.0	0.660 ***	0.031
	2009	72.7	0.659 ***	0.031
	2010	75.2	0.685 ***	0.025
	2011	71.4	0.666 ***	0.029
	2012	61.1	0.604 ***	0.043
	2013	52.1	0.590 ***	0.046
	2014	49.0	0.596 ***	0.045
	2015	50.3	0.616 ***	0.040
	2016	48.2	0.596 ***	0.045
	2017	48.0	0.604 ***	0.043
	2018	51.2	0.622 ***	0.039
	2019	79.4	0.717 ***	0.018
	2020	55.4	0.629 ***	0.038

SE, standard error; (ref.) denotes the reference variable omitted to prevent multi-collinearity. References were selected due to being the most frequently observed in the dataset. Country Income Group refers to the income group classification of the affected country. Year denotes the year the disaster event occurred.
+ Coefficients are given as marginal effects calculated at the mean.
* p < 0.1; ** p < 0.05; *** p < 0.01
