# Supplementary material for: Human and economic impacts of natural disasters: can we trust the global data?
Source: Sci Data. 2022 Sep 16;9:572. doi: 10.1038/s41597-022-01667-x (PMC9481555; doi:10.1038/s41597-022-01667-x)
Supplement: Supplementary file 3 — Results of logistic regression analysis to test associations between the probability of data to be missing on select human loss variables and observable data in the Emergency Events Database (EM-DAT) [file 41597_2022_1667_MOESM3_ESM.pdf]

| | | No. of Affected | | | No. of Missing | | | No. of Deaths | | |
|---|---|---|---|---|---|---|---|---|---|---|
| | **Observations (n)** | 11,024 | | | 4,551 | | | 11,024 | | |
| | **Pseudo $R^2$** | 0.206 | | | 0.621 | | | 0.188 | | |
| **Observable Characteristic** | **Variable** | **% of missing data** | **Coefficient +** | **SE** | **% of missing data** | **Coefficient +** | **SE** | **% of missing data** | **Coefficient +** | **SE** |
| Country Income Group | Low-income | 17.9 | - 0.089 *** | 0.008 | 15.8 | 0.001 | 0.233 | 12.9 | - 0.031 *** | 0.005 |
| | Lower-middle-income | 18.5 | - 0.103 *** | 0.007 | 15.1 | - 0.005 | 0.226 | 9.1 | - 0.052 *** | 0.005 |
| | Upper-middle-income | 16.6 | - 0.121 *** | 0.007 | 13.8 | - 0.007 | 0.299 | 13.9 | - 0.027 *** | 0.005 |
| | High-income (ref.) | 36.2 | - | - | 15.1 | - | - | 16.6 | - | - |
| Natural Disaster Type | Drought | 15.8 | 0.066 *** | 0.025 | 10.3 | 0.138 | 4.877 | 44.0 | 0.338 *** | 0.030 |
| | Earthquake | 17.5 | 0.067 *** | 0.018 | 15.0 | 0.025 | 1.001 | 15.1 | 0.019 ** | 0.010 |
| | Epidemic | 10.6 | 0.019 | 0.015 | 9.7 | 0.161 | 5.565 | 4.1 | - 0.051 *** | 0.006 |
| | Extreme Temperature Event | 57.1 | 0.447 *** | 0.028 | 13.8 | 0.038 | 1.518 | 9.1 | - 0.039 *** | 0.006 |
| | Flood (ref.) | 14.5 | - | - | 16.3 | - | - | 13.4 | - | - |
| | Landslide | 37.8 | 0.343 *** | 0.027 | 12.5 | - 0.019 | 0.813 | 1.5 | - 0.072 *** | 0.005 |
| | Storm | 31.2 | 0.152 *** | 0.012 | 16.4 | 0.002 | 0.073 | 13.1 | - 0.004 | 0.005 |
| | Volcanic Activity | 7.2 | - 0.052 * | 0.031 | 3.6 | - 0.021 | 0.895 | 32.4 | 0.190 *** | 0.041 |
| | Wildfire | 28.3 | 0.149 *** | 0.031 | 14.6 | 0.038 | 1.493 | 13.4 | 0.002 | 0.013 |
| | Other disasters | 36.8 | 0.431 *** | 0.079 | 5.3 | 0.003 | 0.120 | 29.8 | 0.160 *** | 0.060 |
| Year | 1990 (ref.) | 23.1 | - | - | 0 | - | - | 13.9 | - | - |
| | 1991 | 3.0 | - 0.139 *** | 0.009 | 0 | Omitted § | | 1.9 | - 0.068 *** | 0.006 |
| | 1992 | 5.2 | - 0.123 *** | 0.012 | 0.43 | 0.984 | 1.395 | 3.9 | - 0.057 *** | 0.008 |
| | 1993 | 6.4 | - 0.117 *** | 0.012 | 0.37 | 0.985 | 1.297 | 4.5 | - 0.051 *** | 0.009 |
| | 1994 | 10.2 | - 0.079 *** | 0.019 | 0 | Omitted § | | 5.1 | - 0.047 *** | 0.010 |
| | 1995 | 7.6 | - 0.110 *** | 0.014 | 0 | Omitted § | | 5.1 | - 0.050 *** | 0.009 |
| | 1996 | 15.0 | - 0.029 | 0.026 | 0 | Omitted § | | 7.3 | - 0.023 | 0.015 |
| | 1997 | 10.8 | - 0.071 *** | 0.019 | 0 | Omitted § | | 4.6 | - 0.051 *** | 0.009 |
| | 1998 | 6.6 | - 0.112 *** | 0.012 | 0 | Omitted § | | 6.1 | - 0.041 *** | 0.010 |
| | 1999 | 6.5 | - 0.106 *** | 0.013 | 0 | Omitted § | | 1.7 | - 0.070 *** | 0.005 |
| | 2000 | 3.8 | - 0.134 *** | 0.009 | 0 | Omitted § | | 2.5 | - 0.065 *** | 0.006 |
| | 2001 | 2.2 | - 0.148 *** | 0.007 | 0 | Omitted § | | 1.6 | - 0.070 *** | 0.005 |
| | 2002 | 3.6 | - 0.135 *** | 0.009 | 0 | Omitted § | | 1.9 | - 0.069 *** | 0.005 |
| | 2003 | 31.6 | 0.124 *** | 0.037 | 0.26 | 0.989 | 1.422 | 18.6 | 0.055 ** | 0.025 |
| | 2004 | 31.1 | 0.112 *** | 0.036 | 0 | Omitted § | | 28.4 | 0.130 *** | 0.034 |
| | 2005 | 36.8 | 0.185 *** | 0.039 | 0.2 | 0.990 | 1.531 | 24.8 | 0.103 *** | 0.030 |
| | 2006 | 28.0 | 0.133 *** | 0.037 | 0 | Omitted § | | 25.8 | 0.119 *** | 0.032 |
| | 2007 | 33.4 | 0.168 *** | 0.039 | 0 | Omitted § | | 19.8 | 0.071 *** | 0.026 |
| | 2008 | 30.5 | 0.139 *** | 0.038 | 0 | Omitted § | | 24.0 | 0.099 *** | 0.030 |
| | 2009 | 31.5 | 0.140 *** | 0.039 | 0.26 | 0.988 | 1.433 | 25.8 | 0.130 *** | 0.035 |
| | 2010 | 34.2 | 0.165 *** | 0.039 | 0 | Omitted § | | 25.3 | 0.116 *** | 0.032 |
| | 2011 | 31.2 | 0.147 *** | 0.040 | 0 | Omitted § | | 23.6 | 0.098 *** | 0.031 |
| | 2012 | 29.4 | 0.076 ** | 0.034 | 0 | Omitted § | | 22.3 | 0.085 *** | 0.029 |
| | 2013 | 26.1 | 0.072 ** | 0.034 | 0.28 | 0.988 | 1.355 | 21.3 | 0.073 *** | 0.028 |
| | 2014 | 33.6 | 0.148 *** | 0.041 | 53.0 | 0.991 | 0.789 | 17.1 | 0.042 * | 0.024 |
| | 2015 | 33.6 | 0.145 *** | 0.040 | 81.2 | 0.993 | 0.678 | 8.0 | - 0.024 * | 0.013 |
| | 2016 | 37.1 | 0.208 *** | 0.045 | 82.6 | 0.992 | 0.723 | 10.0 | - 0.003 | 0.017 |
| | 2017 | 33.4 | 0.119 *** | 0.039 | 74.8 | 0.992 | 0.749 | 9.6 | - 0.004 | 0.017 |
| | 2018 | 37.8 | 0.183 *** | 0.045 | 83.6 | 0.990 | 0.792 | 11.8 | 0.004 | 0.019 |
| | 2019 | 37.6 | 0.206 *** | 0.043 | 77.1 | 0.993 | 0.628 | 4.0 | - 0.046 *** | 0.001 |
| | 2020 | 26.8 | 0.085 ** | 0.036 | 77.7 | 0.992 | 0.703 | 8.0 | - 0.017 | 0.015 |

SE, standard error; ref. denotes the reference variable omitted to prevent multi-collinearity. References were selected due to being the most frequently observed in the dataset. Country Income Group refers to the income group classification of the affected country. Year denotes the year the disaster event occurred.
+ Coefficients are given as marginal effects calculated at the mean.
* $p < 0.1$; ** $p < 0.05$; *** $p < 0.01$
§ Variable was omitted by STATA due to perfectly predicting the probability to be missing.
