# Supplementary material for: Human and economic impacts of natural disasters: can we trust the global data?
Source: Sci Data. 2022 Sep 16;9:572. doi: 10.1038/s41597-022-01667-x (PMC9481555; doi:10.1038/s41597-022-01667-x)
Supplement: Supplementary file 5 — Reference List [file 41597_2022_1667_MOESM5_ESM.docx]

**Reference List**

The reference list provided below corresponds to the references cited within Supplementary Table 3. For continuity, the reference numbering is consistent with that used in the article main text.

19. Brooks, N., Adger, W. N. & Kelly, P. M. The determinants of vulnerability and adaptive capacity at the national level and the implications for adaptation. *Glob. Environ. Chang.* **15**, 151–163 (2005).

20. Dilley, M., Chen, R. S., Deichmann, U., Lerner-Lam, A. L. & Arnold, M. Natural Disaster Hotspots: A Global Risk Analysis. *World Bank* https://openknowledge.worldbank.org/handle/10986/7376 (2005).

21. Barredo, J. I. Major flood disasters in Europe: 1950-2005. *Nat. Hazards.* **42**, 125–148 (2007).

22. Doocy, S., Daniels, A., Murray, S. & Kirsch, T. D. The Human Impact of Floods: a Historical Review of Events 1980-2009 and Systematic Literature Review. *PLoS Curr.* **5**, (2013).

23. Yang, D. Coping with disaster: The impact of hurricanes on international financial flows, 1970-2002. *B.E. J. Econ. Anal. Policy.* **8**, (2008).

33. Lesk, C., Rowhani, P. & Ramankutty, N. Influence of extreme weather disasters on global crop production. *Nature.* **529**, 84–87 (2016).

34. Kahn, M. E. The Death Toll from Natural Disasters: The Role of Income, Geography, and Institutions. *Rev. Econ. Stat.* **87**, 271–284 (2005).

35. Noy, I. The macroeconomic consequences of disasters. *J. Dev. Econ.* **88**, 221–231 (2009).

36. Alcántara-Ayala, I. Geomorphology, natural hazards, vulnerability and prevention of natural disasters in developing countries. *Geomorphology.* **47**, 107–124 (2002).

37. Toya, H. & Skidmore, M. Economic development and the impacts of natural disasters. *Econ. Lett.* **94**, 20–25 (2007).

38. Jonkman, S. N. Global Perspectives on Loss of Human Life Caused by Floods. *Nat. Hazards.* **34**, 151–175 (2005).

39. Strömberg, D. Natural Disasters, Economic Development, and Humanitarian Aid. *J. Econ. Perspect.* **21**, 5–222 (2007).

40. Kovács, G. & Spens, K. Identifying challenges in humanitarian logistics. *Int. J. Phys. Distrib. Logist. Manag.* **39**, 506–528 (2009).

41. Barredo, J. I. Normalised flood losses in Europe: 1970-2006. *Nat. Hazards Earth Syst. Sci.* **9**, 97–104 (2009).

42. Nadim, F., Kjekstad, O., Peduzzi, P., Herold, C. & Jaedicke, C. Global landslide and avalanche hotspots. *Landslides.* **3**, 159–173 (2006).

43. Kellenberg, D. K. & Mobarak, A. M. Does rising income increase or decrease damage risk from natural disasters? *J. Urban Econ.* **63**, 788–802 (2008).

44. Deressa, T., Hassan, R. M. & Ringler, C. *Measuring Ethiopian farmers’ vulnerability to climate change across regional states*. IFPRI Discussion Paper 806 (International Food Policy Research Institute, 2008).

45. Raschky, P. A. Institutions and the losses from natural disasters. *Nat. Hazards Earth Syst. Sci.* **8**, 627–634 (2008).

46. Mohapatra, S., Joseph, G. & Ratha, D. Remittances and natural disasters: Ex-post response and contribution to ex-ante preparedness. *Environ. Dev. Sustain.* **14**, 365–387 (2012).

47. Kvaløy, B., Finseraas, H. & Listhaug, O. The publics’ concern for global warming: A cross-national study of 47 countries. *J. Peace Res*. **49**, 11–22 (2012).
