# Supplementary material for: Human and economic impacts of natural disasters: can we trust the global data?
Source: Sci Data. 2022 Sep 16;9:572. doi: 10.1038/s41597-022-01667-x (PMC9481555; doi:10.1038/s41597-022-01667-x)
Supplement: Supplementary file 6 — Glossary of conventional and advanced missing data methods [file 41597_2022_1667_MOESM6_ESM.pdf]

Method	Description	Notes
**Conventional methods**		
Column deletion	Deleting variables which have a high proportion of missing data. A threshold of greater than 60% missing data is commonly suggested.	This method should only be considered for variables which are not necessary to the analysis.
Complete Case Analysis (CCA) (Listwise deletion)	Also referred to as row deletion. Observations with missing data on at least one variable of interest are excluded.	CCA is used by default in most statistical software programmes.<br>It yields a complete dataset which facilitates the use of conventional data analysis methods.<br>When a dataset contains a large proportion of missing data, CCA excludes a large fraction of the original data and reduces the statistical power of analyses.<br>CCA relies on the assumption that missing data are MCAR or MAR if all predictors of missingness are included in the analysis.
Aggregating data	Compiling and expressing individual-level data into summary forms for statistical analysis.	Missing data are masked within summary statistics, minimising their relative impact. However, the precision of analyses are substantially reduced.
Dummy variable adjustment	For continuous variables, a dummy variable is created to indicate if data is missing on that variable. For categorical variables, an additional category is created to hold cases with missing data.	This method allows the entire dataset to be used in data analysis, maximising the sample size and statistical power. However, dummy variable adjustment has been shown to yield biased parameter estimates.
Available Case Analysis (ACA)<br>(Pairwise deletion)	All observed values for each variable or pair of variables are utilised to calculate sample 'moments' (population mean, variance etc.). In other words, only missing data for the variable, or pairs of variables of interest are excluded. Sample moments are then included in the data analysis in place of population parameters.	Like CCA, this method yields a complete dataset which facilitates the use of conventional data analysis methods.<br>As ACA uses all the data available for each analysis, it does not skew summary statistics.<br>For bivariate and multivariate analyses, ACA requires sufficient correlation between variables to yield consistent parameter estimates. However, as different subsets of the data are used to calculate sample moments, there is no guarantee of this.<br>ACA relies on the assumption of MCAR.
Mean imputation	Missing values are substituted with a single unconditional mean of the observed values.	Single imputation methods yield a complete dataset and facilitate the use of conventional data analysis methods independently of missing data methods.<br>As with most single imputation methods, mean imputation yields biased parameter estimates.<br>Predicted values do not contain random error, so sample variation is reduced. This can lead to an underestimation of standard errors and optimistic significance values. This issue is magnified with higher proportions of missing data.
Regression-based imputation	Missing values are substituted with a single, predicted value estimated using regression methods, conditional on observed predictors of missingness.	Single imputation methods yield a complete dataset and facilitate the use of conventional data analysis methods independently of missing data methods.<br>Relies on the assumption that missing data are MAR.<br>As with mean imputation, regression-based imputation yields biased parameter estimates and uncertainty in the predicted value is not adequately reflected.<br>Predicted values do not contain random error, so sample variation is reduced. This can lead to an underestimation of standard errors and optimistic significance values. This issue is magnified with higher proportions of missing data.
Data merging	Merging data sources, or data subsets by integration or aggregation to supplement existing data.	Data merging by conditional merging is most appropriate when merging incomplete datasets. This involves filling missing data gaps with observed values found in other source datasets.<br>Data loss and file-matching errors can occur if there is heterogeneity in the coding of data across datasets, or if there is heterogeneity in the number and type of variables. Hence, datasets need to be standardised prior to merging. Data matching is also necessary to prevent the duplication of data across datasets. This method can therefore be time-consuming.
**Advanced methods**		
Inverse probability weighting (IPW)	'Complete cases' are weighted by the inverse probability of being observed. Weights are calculated using a binary regression model conditional on observed predictors of missingness.	IPW rebalances the data so complete cases better represent the entire sample.<br>By adjusting for missing data without manipulating the full dataset, IPW does not create issues of incompatibility with subsequent data analysis.<br>Relies on the assumption that missing data are MCAR or MAR, if all predictors of missingness are included in the binary regression model.
Maximum-likelihood	Uses all observed data to generate the parameter estimates most likely to result from the available data. Likelihoods are computed separately for observations with complete and incomplete data on the variables of interest. The product of the individual likelihoods is then maximised to give the maximum-likelihood parameter estimates.	Maximum likelihood yields asymptotically unbiased and efficient parameter estimates.<br>Missing data and parameter estimation are handled in a single step. However, this requires all predictors of missingness to be specified in the intended analysis model.<br>Relies on the assumption that missing data are MAR but can be modified for missing data which are MNAR.<br>For each variable with missing data, parametric models for the joint distributions need to be specified. This is potentially difficult and parameter estimates may be sensitive to the choice of model.<br>Maximum-likelihood is limited to only linear models and requires specialist statistical software packages.
Multiple imputation	An extension of regression-based single imputation. Multiple imputation involves 3 steps:<br>1. Imputation using regression methods is performed several times, generating *m* imputed datasets. Each dataset contains a different, randomly drawn, imputed value for all missing values.<br>2. Datasets are analysed separately using standard methods.<br>3. The parameter estimates and standard errors obtained from each are combined using Rubin's Rules to generate a single set of parameter estimates and standard errors.	Multiple imputation yields asymptotically unbiased and efficient parameter estimates.<br>By generating multiple, randomly drawn imputed values, multiple imputation adequately accounts for uncertainty in the predicted value.<br>Makes no assumptions about the missing data mechanism; can be modified for missing data which is MNAR.<br>Requires several decisions to be made on: the type of imputation model, the number of imputations (*m*), the number of iterations between imputations and the choice of prior distribution. With larger proportions of missing data, a greater number of imputations are required. Generally, $m = 20$ is considered sufficient.<br>Potentially computationally difficult with a large number of variables and/or observations.
**Other advanced methods**		
Hot deck imputation	Each missing value is replaced with a plausible, observed value taken from similar observations within the same classification. Imputed values may be selected at random, or by using distance metrics, such as nearest neighbour matching.	This method yields a complete dataset and facilitates the use of conventional data analysis methods independently of missing data methods.<br>Hot deck imputation does not require missing values to be modelled. Therefore, parameter estimates are less sensitive to model misspecifications.<br>If there are large proportions of missing data, only a small sample of observations may be used to impute missing values, leading to replication of values and reduced sample variation. This can lead to an underestimation of standard errors and optimistic significance values.
Bayesian simulation	An extension of multiple imputation. Missing data are treated as additional, unknown variables for which posterior predictive distributions can be calculated by specifying a missing data model and Bayesian priors. Algorithms, such as Monte Carlo Markov Chain are then used to yield parameter estimates from the posterior predictive distributions.	Missing data and parameter estimation are handled in a single step.<br>Bayesian analysis can be easily adapted for incomplete data.<br>Bayesian priors may be based on expert opinion which can improve the reliability of results.<br>As with multiple imputation, Bayesian simulation adequately accounts for uncertainty due to the missing values.<br>Can be modified to account for any assumption on the mechanism of missing data<br>Parameter estimates may be sensitive to model misspecifications.<br>Requires specialist software and can be highly complex.

ACA, available case analysis; CCA, complete case analysis; MAR, missing at random; MCAR, missing completely at random; MNAR, missing not at random.
