# Supplementary material for: Human and economic impacts of natural disasters: can we trust the global data?
Source: Sci Data. 2022 Sep 16;9:572. doi: 10.1038/s41597-022-01667-x (PMC9481555; doi:10.1038/s41597-022-01667-x)
Supplement: Supplementary file 7 — STATA code [file 41597_2022_1667_MOESM7_ESM.pdf]

```
* 25/03/2022
*** Missing data ***

. use "/Users/Documents/UCLouvain/EM-DAT/Missing data/Short
piece/Analysis/clean(±5%)EM-DAT_natural_max_22:06:2021.dta


** Variables **

* Dependent variable *

gen missing_totaldamages = 0
replace missing_totaldamages = 1 if missing(totaldamages)
* check *
mdesc totaldamages if missing_totaldamages==0

clonevar yE= missing_totaldamages
label var yE "the probability of total estimated damages being missing"
tab yE


* Independent variables *

* Disaster severity proxy *
* log totaldeaths
gen logtotaldeaths = log(totaldeaths)
label var logtotaldeaths "log transformation of the no. of total deaths"
summ logtotaldeaths

* income group*
gen low_income = (incomegroup==1) if !missing(incomegroup)
gen lowmiddle_income = (incomegroup==2) if !missing(incomegroup)
gen uppermiddle_income = (incomegroup==3) if !missing(incomegroup)
gen high_income = (incomegroup==4) if !missing(incomegroup)

global di "low_income lowmiddle_income uppermiddle_income"
* where high-income is omitted as the reference

* year
levelsof year, local(year)
foreach y of local year {
	gen d`y' = (year==`y') if !missing(year)
}

global dy "d1991 d1992 d1993 d1994 d1995 d1996 d1997 d1998 d1999 d2000
d2001 d2002 d2003 d2004 d2005 d2006 d2007 d2008 d2009 d2010 d2011 d2012
d2013 d2014 d2015 d2016 d2017 d2018 d2019 d2020"
* where 1990 is omitted as the reference

* disaster type
```

```
gen other_dis = (ctype==1) if !missing(ctype)
gen drought = (ctype==3) if !missing(ctype)
gen earthquake = (ctype==4) if !missing(ctype)
gen epidemic = (ctype==5) if !missing(ctype)
gen extremetemp = (ctype==6) if !missing(ctype)
gen flood = (ctype==7) if !missing(ctype)
gen landslide = (ctype==12) if !missing(ctype)
gen storm = (ctype==15) if !missing(ctype)
gen volcanic = (ctype==17) if !missing(ctype)
gen wildfire = (ctype==18) if !missing(ctype)

global dt "drought earthquake epidemic extremetemp landslide storm volcanic wildfire
other_dis"
* where flood is omitted as the reference

** Diagnosis **

** Proportion of missing data **
asdoc mdesc id year seq ISO type subgroup disgroup start_* end_* incomegroup
affected missing deaths totaldeaths reconstr_costs insured_damages totaldamages
*_affected ind* dir* houses_* commercial_* bridges_* roads_* health_* forest_*
farm_* edu_* rails_*


** Patterns of missing data **
misspattern reconstr_costs insured_damages totaldamages affected missing deaths
totaldeaths, novarsort indivsname(disasters) label ytitle("11,124 disasters")
legend(position(6) cols(2))
graph export "final2_misspattern.pdf", replace


** Logistic regression **

* total esimated damages *
 cd "/Users/beckyjones/Documents/UCLouvain/EM-DAT/Missing data/Short
piece/Analysis/Logistic regression"
capture log, close
log using "totaldamages.log", replace

putdocx clear
putdocx begin
putdocx paragraph, style(Title)
putdocx text ("Logistic regression")
putdocx textblock end

logistic yE logtotaldeaths $di $dt $dy
putdocx table logistic=etable, title(Logistic_totaldamages)

mfx
estat classification
```

```
log close
putdocx save Logisticregression_damages, replace
docx2pdf Logisticregression_damages, replace


* Proportions of missing data by cateogorical independent variables
by incomegroup, sort: tab missing_totaldamages
by ctype, sort: tab missing_totaldamages
by year, sort: tab missing_totaldamages


** Robustness checks **

* Probit *
 cd "/Users/beckyjones/Documents/UCLouvain/EM-DAT/Missing data/Short piece/Analysis/Robustness checks/Probit"
capture log, close
log using "probit_totaldamages.log", replace

putdocx clear
putdocx begin
putdocx paragraph, style(Title)
putdocx text ("Probit")
putdocx textblock end

probit yE logtotaldeaths $di $dt $dy
putdocx table probit=etable, title(Probit_totaldamages)

mfx
estat classification

log close
capture log, close
log using "probit_totaldeaths.log", replace

probit ytH logtotaldamages $di $dt $dy
putdocx table probit=etable, title(Probit_totaldeaths)

mfx
estat classification

log close
putdocx save Probit, replace
docx2pdf Probit, replace


* Conditional logit *
```

```
. cd "/Users/beckyjones/Documents/UCLouvain/EM-DAT/Missing data/Short
piece/Analysis/Conditional logit/Economic"
capture log, close
log using "Conditional_economic.log", replace

xtset year
xtlogit yE logtotaldeaths $di $dt, fe

margins, dydx(logtotaldeaths $di $dt) atmeans

log close

. cd "/Users/beckyjones/Documents/PhD/EM-DAT/Missing data/Short
piece/Analysis/Conditional logit/Health"
capture log, close
log using "Conditional_health.log", replace

xtlogit ytH logtotaldamages $di $dt, fe

margins, dydx(logtotaldamages $di $dt) atmeans

log close


** Additional variables

gen missing_affected = 0
replace missing_affected = 1 if missing(affected)
* check *
mdesc affected if missing_affected==0

clonevar yA= missing_affected
label var yA "the probability of no. of people affected to be missing"
tab yA

gen missing_missing = 0
replace missing_missing = 1 if missing(missing)
* check *
mdesc missing if missing_missing==0

clonevar yM= missing_missing
label var yM "the probability of no. of people missing to be missing"
tab yM

gen missing_deaths = 0
replace missing_deaths = 1 if missing(deaths)
* check *
mdesc deaths if missing_deaths==0

clonevar yD= missing_deaths
```

```
label var yD "the probability of no. of deaths to be missing"
tab yD

* Disaster severity proxy *
* log totaldamages
gen logtotaldamages = log(totaldamages)
label var logtotaldamages "log transformation of the no. of total damages"
summ logtotaldamages


** Logistic regression **
* no. affected *
 cd "/Users/beckyjones/Documents/UCLouvain/EM-DAT/Missing data/Short
piece/Analysis/Logistic regression"
capture log, close
log using "affected.log", replace

putdocx clear
putdocx begin
putdocx paragraph, style(Title)
putdocx text ("Logistic regression - additional variables")
putdocx textblock end

logistic yA $di $dt $dy
putdocx table logistic=etable, title(Logistic_affected)
putdocx pagebreak

mfx
estat classification
log close

capture log, close
log using "missing.log", replace

logistic yM $di $dt $dy
putdocx table logistic=etable, title(Logistic_missing)

mfx
estat classification
log close

capture log, close
log using "deaths.log", replace

logistic yD $di $dt $dy
putdocx table logistic=etable, title(Logistic_deaths)

mfx
estat classification
log close
```

```
putdocx save Logisticregression_additional, replace
docx2pdf Logisticregression_additional, replace

* Varying the functional form of year

gen year2 = year^2
logistic yE logtotaldeaths year year2 $di $dt
logistic yA year year2 $di $dt
logistic yM year year2 $di $dt
logistic yD year year2 $di $dt


* Proportions of missing data by categorical independent variables
* No. of people affected
by incomegroup, sort: tab missing_affected
by ctype, sort: tab missing_affected
by year, sort: tab missing_affected

* No. of people missing
by incomegroup, sort: tab missing_missing
by ctype, sort: tab missing_missing
by year, sort: tab missing_missing

* No. of deaths
by incomegroup, sort: tab missing_deaths
by ctype, sort: tab missing_deaths
by year, sort: tab missing_deaths

* No. of deaths
. cd "/Users/beckyjones/Documents/PhD/EM-DAT/Missing data/Short
piece/Analysis/Conditional logit/Health"
capture log, close
log using "Conditional_health(deaths).log", replace

xtlogit yH logtotaldamages $di $dt, fe

margins, dydx(logtotaldamages $di $dt) atmeans

log close

label define nsource `s', modify
levelsof nsource, local(nsource)
foreach s of local nsource {
	gen d_`s' = (nsource==`s') if !missing(nsource)
}
```
